# How to Treat COVID-19 Patients at Home in the Italian Context: An Expert Opinion

**DOI:** 10.3390/idr13010028

**Published:** 2021-03-20

**Authors:** Davide Roberto Donno, Ignazio Grattagliano, Alessandro Rossi, Pierangelo Lora Aprile, Gerardo Medea, Erik Lagolio, Guido Granata, Nicola Petrosillo, Claudio Cricelli

**Affiliations:** 1 Clinical and Research Department for Infectious Diseases, National Institute for Infectious Diseases L. Spallanzani IRCCS, 00149 Rome, Italy; davideroberto.donno@inmi.it (D.R.D.); nicola.petrosillo@inmi.it (N.P.); 2 Italian College of General Practitioners and Primary Care, 50142 Florence, Italy; studiomedico@grattagliano.it (I.G.); rossi.alessandro@simg.it (A.R.); lora.aprile.pierangelo@gmail.com (P.L.A.); medea.gerardo@alice.it (G.M.); erik.lagolio@gmail.com (E.L.); cricelli@gmail.com (C.C.)

**Keywords:** COVID-19, home care, SARS-CoV-2, COVID-19 management, general practice, primary care

## Abstract

The impact of the coronavirus disease (COVID-19), caused by the novel coronavirus SARS-CoV-2, continues to be widespread, with more than 100 million cases diagnosed in more than 220 countries since the virus was first identified in January 2020. Although patients with mild to moderate forms of COVID-19 could be efficiently managed at home, thus reducing the pressure on the healthcare system and minimizing socio-psychological impact on patients, no trial has been proposed, conducted, or even published on COVID-19 home therapy to date. These expert opinions provide indications on the therapeutical at home management of COVID-19 patients, based on the evidence from the literature and on current guidelines.

## 1. Introduction

The impact of the coronavirus disease (COVID-19), caused by the novel coronavirus SARS-CoV-2, continues to be widespread, with more than 100 million cases diagnosed in more than 220 countries since the virus was first identified in January 2020 [1]. Most commonly, COVID-19 has the appearance of a flu-like syndrome with a variety of mild symptoms including rhinitis, pharyngitis, cough, and fever. In some unfortunate patients, a severe acute respiratory syndrome and arterial microthromboembolism may occur, thus requiring hospitalization [2].

Although patients with mild to moderate forms of COVID-19 could be efficiently managed at home, thus reducing the pressure on the healthcare system and minimizing socio-psychological impact on patients, no trial has been proposed, conducted, or even published on COVID-19 home therapy to date. Therefore, even though today we know this virus better and the tools to identify the subjects who may develop a more aggressive disease form are clearer, there is a need for addressing unsolved questions on and unmet needs in the pharmaceutical treatment of COVID-19 at home, by using simple and understandable indications to general practitioners/primary care physicians, based on the evidence from the literature and on official recommendations by the Italian Ministry of Health [3] and Istituto Superiore di Sanità [4] that represent the national health agencies in Italy.

The aim of this manuscript is to provide solid suggestions on specific statements raised by general practitioners/primary care physicians on the pharmaceutical treatment of COVID-19 patients at home.

## 2. Materials and Methods

With over 8000 members, the Italian College of General Practitioners and Primary Care (Società Italiana di Medicina Generale e delle Cure Primarie, SIMG) is the main scientific society of family medicine in Italy. The SIMG is regularly accredited by the Ministry of Health and acts as the main promoter for education, training, and research activities for over 5000 general practitioners (GPs).

Many important issues on the approach and management of COVID-19 patients affect GPs. Recently, the SIMG promoted an online survey asking all members to identify the main problems concerning their daily activity with COVID-19 patients at home. The main selected points identified the following statements reported in this article.

A restricted number of two SIMG members designated ad hoc by the President of the SIMG, in collaboration with two infectious disease specialists, joined a non-voting panel group for addressing the statements according to the evidence from the literature or expert opinion.

The expert opinion development process started with eleven different reviews of the literature, one for each question.

The literature search was performed through PubMed and the Cochrane COVID-19 Study Register. The search period was from 1 January 2020 to 8 February 2021.

For each question, recommendations were drafted by non-voting members based on the retrieved literature.

The produced statements were then reviewed by an external panel constituted by other SIMG members and additional infectious disease specialists not previously involved in the statement preparation. The external panel included ten GPs, SIMG members, and ten infectious disease specialists.

A dedicated voting process (collection of voting forms through individual email messages) was developed, owing to the emergency situation and the clinical duties related to COVID-19, which eventually did not allow the organization of meetings with participation of the full voting panel. Inn more detail, voting panel members were provided with the proposed statements. Each voting member was then allowed to individually vote in favor or against each of the statements and to propose possible modifications. For statements with an agreement <75%, further voting rounds were conducted after implementation of dedicated amendments based on the provided comments. After reaching an agreement ≥75% for all the eleven statements, all the authors reviewed and approved the final manuscript.

## 3. Results

### 3.1. Statements

#### 3.1.1. How Can GPs Identify Asymptomatic, Mild, Moderate, or Severe COVID-19 Patients?

According to the WHO, asymptomatic COVID-19 is defined as a person with a positive nucleic acid amplification test (NAAT) for SARS-CoV-2 in the absence of symptoms. Mild symptomatic patients meet the case definition for COVID-19 without evidence of viral pneumonia or hypoxia. Moderate illness may include: clinical signs of pneumonia (fever, cough, dyspnea, fast breathing) but no signs of severe pneumonia, including peripheral oxygen saturation (SpO2) ≥ 90% in ambient air. Severe disease is defined by clinical signs of pneumonia (fever, cough, dyspnea, fast breathing) plus one of the following: respiratory rate >30 breaths/min; severe respiratory distress; SpO2 < 90% in ambient air.

#### 3.1.2. Which COVID-19 Patients Could Be Treated at Home?

The decision as to whether isolate and care for an infected person at home depends on clinical evaluation of the COVID-19 patient and should be made on a case-by-case basis.

Patients who are asymptomatic or those with mild or moderate disease without risk factors for poor outcome, including age >60 years, smoking, obesity, cardiovascular disease, diabetes mellitus, chronic lung disease, chronic kidney disease, immunosuppression, and cancer, may not require emergency interventions or hospitalization, and could be suitable for home isolation and care [5].

#### 3.1.3. How to Manage Fever in COVID-19 Patients at Home?

The WHO recommends that patients with COVID-19 receive treatment for their symptoms, such as antipyretics for fever and pain [5]. Paracetamol is suggested as a safer and recommendable choice for the early and home management of COVID-19 patients [3,5]. Non-steroidal anti-inflammatory drugs (NSAIDs), including acetylsalicylic acid and ibuprofen, could be more valuable in treating flu-like syndromes [3] and have a potential benefit against the COVID-19 inflammatory storm associated with a reduction of the risk of worsening respiratory symptoms [6,7,8]. It is not recommended to exceed the doses for these medicaments in accordance with manufacturers’ instructions, i.e., 3 g daily for paracetamol. Attention must be paid to possible side effects due to anti-inflammatory drugs which, as is well known, can cause renal, hepatic, and gastric damage. It is also recommended, during febrile illness, to monitor an appropriate state of nutrition and hydration.

#### 3.1.4. Which Clinical Parameters Should Be Assessed at Home?

Home pulse oximetry is a safe, non-invasive way to assess oxygen saturation in the blood and can support the early identification of low oxygen levels in patients with initially mild or moderate COVID-19, and can identify individuals in need for oxygen therapy or hospitalization [9,10,11]. The respiratory rate is another parameter that is easy to assess at home. Tachypnea is a term used to define rapid and shallow breathing, which should not be confused with hyperventilation that occurs when a patient breathing is rapid but deep. Tachypnea in adults means breathing more than 20 times per minute, considering that 10–20 breaths per minute is the normal range [12].

#### 3.1.5. When Should a COVID-19 Patient Be Referred to the Hospital?

Peripheral oxygen saturation higher than 92% (SpO2 > 92%), measured by pulse oximetry in ambient air, is considered as a safety cut-off for managing a COVID-19 patient at home [3]. Oxygen therapy is recommended when the respiratory rate exceeds 20/minute and SpO2 is equal to or below 92% or 90% in the presence of chronic obstructive pulmonary disease (COPD) patients, in ambient air.

Hospitalization is necessary when vital signs become unstable but also when SpO2 decreases rapidly, i.e., within 2 h [10]. Patients poorly responsive to O2 administration should be urgently considered for hospitalization, if feasible [13]. The decision to hospitalize a COVID-19 patient also depends on the impossibility to provide adequate home assistance because of unhelpful social–familial conditions.

#### 3.1.6. Which Antiviral Treatment Could Be Given for Mild to Moderate COVID-19 Patients at Home?

Antiviral medication is not recommended to be administered at home [3]. Given the evidence of ineffectiveness and many warnings about major side effects, lopinavir/ritonavir is not recommended to treat patients with COVID-19 [14].

The only antiviral for which there is some evidence for its efficacy against COVID-19 is remdesivir, an inhibitor of the viral RNA-dependent RNA polymerase with in vitro inhibitory activity against SARS-CoV-1 and Middle East respiratory syndrome caused by MERS-CoV.

Remdesivir is indicated for COVID-19 patients with pneumonia receiving oxygen, but not requiring high-flow oxygen or non-invasive mechanical ventilation or mechanical ventilation or extracorporeal membrane oxygenation (ECMO) [15]. Remdesevir is therefore not suitable for at home care and should be reserved for hospitalized patients with pulmonary involvement in the early stage of disease.

#### 3.1.7. Can Hydroxycloroquine or Cloroquine Be Given for at Home Treatment of COVID-19 Patients?

Hydroxychloroquine is an anti-inflammatory medication used against rheumatic disorders; cloroquine is an anti-malaria medication used to prevent and treat malaria in areas where malaria remains sensitive to chloroquine.

Some authors have suggested the use of hydroxycloroquine or cloroquine for the home prevention or early treatment of COVID-19 patients [16]. However, in a recent systemic review and meta-analysis of randomized controlled trials, no evidence of hydroxycloroquine or cloroquine effectiveness was found [17]; on the contrary, adverse events following administration, including QT wave prolongation and macular degeneration, have been described [18]. Therefore, currently, there is no indication on at home treatment with hydroxycloroquine or cloroquine for COVID-19 patients.

#### 3.1.8. Is Antithrombotic Prophylaxis Suitable for COVID-19 Patients at Home?

On April 2020, the Italian Drug Agency (AIFA) included low molecular weight heparin (LMWH) among the drugs available for the treatment of COVID-19 patients [19].

COVID-19 is a particularly debilitating illness, even for patients with mild symptoms, therefore, patients are often bedridden for several weeks, with a higher risk for thromboembolic events. Heparin may protect the endothelium, likely decreasing the level of inflammatory biomarkers, and may prevent micro- and macrocirculatory lung dysfunction and possibly limit organ damage [20,21,22]. Therefore, bedridden COVID-19 patients with acute respiratory symptoms could be treated with LMWH at home to prevent pulmonary thromboembolism. A single daily subcutaneous injection of enoxaparin at the prophylactic dose is recommended until the patient recovers mobility: 80 mg/day (8000 IU) in patients with normal renal function and normal body weight (45–100 kg) or mild to moderate chronic kidney disease (CKD) (>30 mL/minute/1.73 m^2^); 100 mg/day (10,000 IU) in patients with high body weight (>100 kg); 40 mg/day (4000 IU) in patients with moderate-to-severe CKD (≤15–30 mL/min/1.73 m^2^) or low body weight (<45 kg) [23,24].

#### 3.1.9. When Could Steroids Be Given at Home to COVID-19 Patients?

Since the most severe forms of COVID-19 are the result of immune system overreaction to the virus itself, including cytokine storm and multiorgan failure, the use of medications able to lower inflammation may produce important benefits in terms of disease control and even final recovery. Dexamethasone, a well-known corticosteroid, demonstrated a reduction in COVID-19 deaths by one-third in patients on ventilators and by one-fifth in those on oxygen [25].

However, patients who are at an earlier stage of the infection may be disadvantaged by using steroids that potentially cause a delay in the clearance of the virus and impair lymphocyte proliferation [26]. Corticosteroid use should be confined only to patients with severe lung dysfunction requiring respiratory assistance, often combined with other remedies [27].

The WHO recommends the use of steroids in COVID-19 only for severe and critical illness and it is opposed to its use in non-severe COVID-19 patients [28]. Most COVID-19 patients at home are non-severe, therefore, the use of steroids at home is limited.

#### 3.1.10. When Could GPs Give Antibiotics to COVID-19 Patients at Home?

Since the beginning of the COVID-19 epidemic, antibiotics with immunomodulatory properties, such as azythromicin, have been proposed for the early treatment of this infection. However, antibiotic therapy for the treatment of a viral infection is ineffective and not recommended. Evidence from the literature does not endorse azithromycin’s widespread use in the treatment of COVID-19 [29,30].

In a recent meta-analysis, bacterial co-infection was identified in 3.5% of COVID-19 patients requiring hospital admission; therefore, the majority of COVID-19 patients at hospital admission may not require empirical antibacterial treatment [31].

Moreover, for COVID-19 patients, widespread use of antibiotics should be discouraged, as it may lead to higher bacterial resistance rates, which will impact the burden of disease and deaths in a population during the COVID-19 pandemic and beyond [32]. In conclusion, antibiotics should not be prescribed at home unless there is a strong clinical suspicion of a bacterial superinfection during the course of COVID-19, as evidenced by a reappearance of fever after a period of defervescence, and/or radiological evidence of new onset pneumonia and/or microbiological evidence of bacterial infection. Most of these episodes occur during long stays in hospital, especially in intensive care and during mechanical ventilation [33].

Only in the presence of a strong suspicion of bacterial superinfection should at home patients receive antibiotics, according to the guidelines for the treatment of community-acquired pneumonia [34].

#### 3.1.11. Nutritional Supplements: Are They Effective for Preventing or Treating COVID-19?

Nutritional supplements have been proposed as potentially useful against SARS-CoV-2, but few have been clinically established.

Lactoferrin is an iron-binding glycoprotein of the transferrin family found in most body fluids, with anti-inflammatory and immunomodulatory properties [35]. Various studies have proposed its use as prophylaxis or as therapy of COVID-19 [36]. However, no evidence from clinical trials is available on its efficacy in the prevention and/or treatment of COVID-19 to date.

Observational studies report consistent independent associations between low serum concentrations of 25-hydroxyvitamin D (the major circulating vitamin D metabolite) and susceptibility to, or mortality from, acute respiratory tract infection, including COVID-19 [37,38]. For this reason, some authors suggested vitamin D use in preventing or even treating COVID-19 [39]. Nevertheless, a recent preprinted, multicenter, double-blind, randomized controlled trial showed no benefit from its administration in this setting [40]. To date, no strong evidence for advocating vitamin D use in COVID-19 treatment and/or prophylaxis is available [41].

## 4. Discussion

The current pandemic of COVID-19 is causing high pressure on healthcare systems, including hospital settings and intensive care units; therefore, there is a need for limiting patient access to hospitals only to COVID-19 individuals for which hospital care could provide a clear benefit in terms of clinical outcome.

During the early phase of COVID-19, patients are usually not seriously ill but present a variety of symptoms, including fever, cough, tiredness, shortness of breath, chills, sore throat, headache, musculoskeletal pain, and loss of taste or smell. Non-hospitalized patients require symptomatic treatment as proposed above; in the future, it is likely that new drugs and compounds, including monoclonal antibodies, will be available for reducing the worsening of COVID-19 and the need for hospital admission [42,43].

In the meantime, it is important that GPs monitor patients at home because in the period of day 5 to day 10 from the onset, respiratory impairment is most likely to develop, particularly in elderly patients and patients with preexisting chronic conditions. Moreover, as a general recommendation, GPs should advise patients that their chronic therapy for diabetes, arterial hypertension, and myocardial conditions should not be discontinued [44].

These expert opinions provide indications on the therapeutical at home management of COVID-19 patients, based on the evidence from the literature and on current guidelines. The statements derived from the expert opinions should be acknowledged and applied to the specific context in which the patient lives and inevitably according to the regional regulations. It should also be opportunely emphasized that the decision as to whether to isolate and care for an infected person at home depends on clinical evaluation of the COVID-19 patient and should be made on a case-by-case basis.

Finally, physicians should take into account patient households when choosing between admission and home management. This general rule is even more important when dealing with SARS-CoV-2 infection. Familial and environmental situations, including the supply of materials for hand/respiratory and environmental hygiene, should be checked in order to guarantee proper adhesion to recommendations for home isolation. Furthermore, full compliance from the caregiver/family is mandatory, in order to restrict movements to and from the household.

When suspecting a COVID-19 case, immediate action must be taken in order to protect the family/caregiver from contaminated biological secreta and waste. Furthermore, the physician should instruct the household to perform regular hand and environmental hygiene, promoting physical distancing, facemask use, and ad hoc restroom use for the infected patient, while guaranteeing proper disinfection of the remaining common areas.
